# Audiovisual synchrony perception in observing human motion to music

**DOI:** 10.1371/journal.pone.0221584

**Published:** 2019-08-27

**Authors:** Akira Takehana, Tsukasa Uehara, Yutaka Sakaguchi

**Affiliations:** 1 Department of Mechanical Engineering and Intelligent Systems, Graduate School of Informatics and Engineering, University of Electro-Communications, Chofu, Tokyo, Japan; 2 Research Center for Performance Art Science, University of Electro-Communications, Chofu, Tokyo, Japan; University of Muenster, GERMANY

## Abstract

To examine how individuals perceive synchrony between music and body motion, we investigated the characteristics of synchrony perception during observation of a Japanese Radio Calisthenics routine. We used the constant stimuli method to present video clips of an individual performing an exercise routine. We generated stimuli with a range of temporal shifts between the visual and auditory streams, and asked participants to make synchrony judgments. We then examined which movement-feature points agreed with music beats when the participants perceived synchrony. We found that extremities (e.g., hands and feet) reached the movement endpoint or moved through the lowest position at music beats associated with synchrony. Movement onsets never agreed with music beats. To investigate whether visual information about the feature points was necessary for synchrony perception, we conducted a second experiment where only limited portions of video clips were presented to the participants. Participants consistently judged synchrony even when the video image did not contain the critical feature points, suggesting that a prediction mechanism contributes to synchrony perception. To discuss the meaning of these feature points with respect to synchrony perception, we examined the temporal relationship between the motion of body parts and the ground reaction force (GRF) of exercise performers, which reflected the total force acting on the performer. Interestingly, vertical GRF showed local peaks consistently synchronized with music beats for most exercises, with timing that was closely correlated with the timing of movement feature points. This result suggests that synchrony perception in humans is based on some global variable anticipated from visual information, instead of the feature points found in the motion of individual body parts. In summary, the present results indicate that synchrony perception during observation of human motion to music depends largely on spatiotemporal prediction of the performer’s motion.

## Introduction

The precise harmonization of one’s movements with accompanying music is an important characteristic of an accomplished dancer. Indeed, the degree of synchrony between visual and auditory information strongly contributes to impressions of artistic performance. In this study, we examined the characteristics of audiovisual synchrony perception while participants watched others perform a physical routine set to music.

The perception of synchrony is an important mechanism that links sensory events detected by different sensory organs. A number of well-controlled experiments using simple stimuli (e.g., light flash and click sound) identified factors associated with simultaneous judgment (SJ) and/or temporal-order judgment (TOJ)[1–5]. Although these basic studies revealed essential factors affecting synchrony perception, they used stimuli that were different from natural stimuli encountered in real life. To address this, an increasing number of recent studies have investigated the characteristics of synchrony perception using real-world stimuli [6–15]. For example, Vatakis and Spence [7] asked participants to complete a TOJ task that involved video clips of several different actions (e.g., speech and piano playing) with a range of different stimulus onset asynchrony (SOA) conditions. They found that the just noticeable difference (JND) was about 60 ms, and the point of subjective simultaneity (PSS) was −50–50 ms, depending on the type of action. Eg and Behne [8] conducted a similar experiment to examine the effects of stimulus length and video image clarity in an SJ task. They reported that the temporal integration window (TIW: temporal integration window within which multisensory events are perceptually integrated into a single unit) widened for longer and more dynamic stimuli compared with shorter and more isolated events, while visual clarity had no effect. Long-term musical training [12–14] and videogame training [15] have also been found to enhance sensitivity to audiovisual asynchrony. These previous reports indicate that, compared with simple stimuli, our perceptual system is less sensitive to asynchrony in natural complex stimuli, and that synchrony perception depends on both the nature of the stimuli and experience of the observer. It is possible that the multiple components contained in complex stimuli provide more temporal clues, making it easier to maintain coherence within a wider temporal integration window [7].

Most previous studies on synchrony perception involved with human actions have dealt with cases in which the actions were accompanied by a sound (e.g., hammering, speech, and instrument playing). However, this is not the case in dance performance. During a hammering action, the moment at which the hammer collides with the target (i.e., visual timing) agrees with the moment at which the striking sound is generated (i.e., auditory timing). Thus, the simultaneity of the two types of information can be defined objectively. In a dance performance, in contrast, body movements and music are not directly linked (music is provided by musicians or a music player and body movements are not expected to make a sound). Thus, synchrony during observation of human movement to music cannot be defined objectively, but only subjectively. Thus, the visual (or movement) feature point that is critical to the perception of synchrony with respect to the beats of the music is not clear. Here, we note that in the present article, the term “beat” is used for the meaning of “pulse” or “tactus,” that is, “beat” indicates a basic rhythmic unit allowing listeners to synchronize with music [16–18].

Several recent studies have dealt with this question. Su [19–21] performed an SJ-task experiment wherein a point-light figure corresponding to human bouncing movement was presented with rhythmic percussion sounds. She postulated several possible candidates for the “visual reference point” (e.g., lowest/highest body position and peak velocity) and investigated these by asking participants to complete an SJ task in which the trajectory of bouncing was manipulated (human motion vs. ball motion). She found that although the participants were asked to judge the simultaneity based on the same criterion (i.e., the lowest position vs. percussion sound), the PSS differed between the motion profiles. Specifically, in the human motion condition, optimal synchrony was obtained ~50 ms earlier in the trial. She thus proposed that the point of maximal velocity during downward movement served as the visual reference (note that the maximal velocity point agreed with the lowest point in the ball motion condition, but preceded the lowest point in the human motion condition). Luck and colleagues [22–25] conducted a related series of experiments examining the motion of music conductors. In their experiments, participants were asked to press a key in accordance with the movement of a point-light figure corresponding with a conductor’s finger (or baton). Although this task differs from the SJ task, conducting is closely related to dance performance in that points of body motion serve as communication media. The researchers found that maximal absolute acceleration and maximal velocity were the primary cues (they used the term “visual beats”) that determined the timing of key presses. In addition, a recent study showed that infants could judge synchrony between dance movement and music [26].In the present study, we sought to contribute to the above-mentioned body of work by examining how observers perceive synchrony between music and human body motion. To this end, we used video clips of an individual performing Japanese Radio Calisthenics (i.e., physical exercise to music). This exercise is performed to piano music and consists of thirteen exercises with various body actions including arm extending and swinging, jumping, and body rotations. A Radio Calisthenics program is broadcasted every morning on the radio in Japan, and most Japanese people are familiar with the practice. It is broadly used as warm-up exercise not only in workplaces but also in elementary schools. Radio Calisthenics is not a type of dance performance and no artistic factors are involved in completing the routine. However, we believe that Radio Calisthenics represents a suitable stimulus because we focused on synchrony perception and did not deal with artistic expression in the present study.

We asked participants to perform a synchrony judgment task with a range of different temporal shifts (or SOAs). Instead of a point-light figure, we used a natural image of a person performing the routine as a visual stimulus. We first examined which visual events (or movement feature points) coincided with the beats of the music when people perceived synchrony. Second, we asked whether people could accurately perceive synchrony when the visual stimulus did not contain “critical (or reference) feature points” (i.e., the visual events matched to the beats of the music). Some previous studies showed that visual information around the endpoints (or turning points) of cyclic movement (i.e., possible feature points) were important for visuomotor coordination task [27–30], suggesting that the endpoints give a significant clue to synchrony perception between sound beat and visual motion. Because physical body motion is continuous in time, however, individuals may use an internal model to anticipate the timing of reference feature points from the preceding visual information [31]. Thus, individuals may be able to judge synchrony between music and body motion even if the reference feature points are not visible. To address this, we performed a second experiment using a video clip in which human motion was displayed only within restricted time regions. Finally, we asked why these feature points served as “visual references” when perceiving audiovisual synchrony. We referred to two possible viewpoints regarding this issue. The first involves the perspective of inter-human communication via body motion. People can realize the motor intention of other persons by observing their behavior. Recent theories, such “common coding of action and perception” [32–34] and “mirror system” [35–39] theories, suggests that such function is mediated by the shared neural mechanism serving perception and motor control/planning. Thus, it is plausible that observers in our experiment unconsciously anticipate the motor intention (and/or somatic sensation) of a performer when they view the motion of the performer. Especially, humans innately move the body to the music sound [40,41]: Many studies have shown that not only adults but also infants (and even animals) spontaneously move their bodies to music [42–46]. Specifically, low-frequency sounds (i.e., bass sounds) play a significant role in this motor entrainment [47,48]. In addition, dance-like body movements are often entrained to the specific phase relationship to the sound beats [49–51] and this entrainment was enhanced by vocalization [52] and observing the other person’s performance [53–55]. Furthermore, a recent study showed that people autonomously elicited structured head movements when they performed audio-motor coordination task (i.e., a drumming task) with the conductor’s visual cue [56]. Considering this tight connection between the beat perception and motor action, it seems likely that some neural process involved in this function plays an important role in perceiving synchrony between sound beat and body movement. Taking the views of “mirror system” into consideration, furthermore, such neural process also mediates synchrony perception between sound beat and visually-observed motion of other people.

The second viewpoint involves the coordination of whole body movement or “motor synergy” [57,58]. During natural human actions, multiple body parts often move in a coordinated manner so as to achieve a given motor task. Eventually, it is plausible that the temporal behavior of certain global variables (e.g., center of mass (CoM) of the whole body) should be correlated with that of local body parts because the coordinated motion of body parts is expected to bring some consistent change in such global variables. Thus, we can consider the possibility that observers not only use the movement features of individual body parts *per se*, but also anticipate the behavior of such global variables from the visual information and adopt the associated feature points as reference points. Here, we focus on CoM as such a global variable. Although CoM is a mathematically defined concept and its position and motion cannot be directly observed, people can indirectly know its dynamical behavior using several sensory clues. One is vestibular input: Trunk movement usually brings CoM motion and head motion together, and resultantly, CoM motion must be strongly correlated with head motion. Thus, vestibular input plausibly conveys information on the CoM motion. Besides, many reports pointed out a close relationship between rhythm perception and vestibular sense [40,41,59–63]. For example, vestibular input disambiguates the rhythm perception of ambiguous auditory rhythm patterns [59–61]. Especially, the finding that direct galvanic stimulation of the vestibular system induced this disambiguation without actual head movement suggests an essential role of the vestibular input in rhythm perception [64]. In daily life, most people bob their head to the music and musicians signal the beat timing just by a slight nod. Putting together these arguments, it seems plausible that the vestibular system is involved in perceiving synchrony between sound beats and body movement. Another clue to the CoM motion is the ground reaction force (GRF). According to the Newtonian mechanics, the acceleration of CoM is determined by the external force acting on one’s body, that is, the sum of gravity force and GRF. Since gravity is constant, the temporal change in GRF reflects the dynamics of CoM. Moreover, the GRF can be sensed as a tactile stimulus at the foot sole, and thus, one can estimate the CoM motion based on this tactile sense. To the best of our knowledge, however, no researchers have analyzed the temporal behavior of GRF in individuals performing physical exercise to music. To examine the temporal characteristics of GRF, we measured the GRF in individuals as they performed Radio Calisthenics, and analyzed the temporal relationship with respect to music beats and body motion. Considering the recent finding that the timing of the peak force point (rather than the endpoint) may be the target in a finger tapping task [65], force sensation or intention might be more fundamental to timing perception than kinematic trajectory. Thus, the analysis of GRF associated with music beats might produce useful information regarding the mechanisms of audiovisual synchrony perception during observation of human motion to music.

## Experiment 1

### Methods

#### Participants

Fifteen graduate and undergraduate students (14 men and 1 woman) at the University of Electro-Communications participated in this experiment. They all had experience performing Radio Calisthenics. They were given a pre-paid book voucher (valued at a 1000 Japanese Yen: about 9 US dollars) to compensate them for their participation. All participants were naïve with respect to the purpose of the study, and reported normal hearing and normal or corrected-to-normal vision.

This experiment was approved by the University of Electro-Communications Institutional Review Board for Human Subjects Research (#16032), and was in accordance with the ethical standards in the Declaration of Helsinki. We obtained written informed consent from all participants.

#### Apparatus

The experiment was conducted in a dark experimental booth built in a quiet laboratory room. Participants were seated at a desk on which a response keyboard was placed. Visual and auditory stimuli were generated using experiment control software (Presentation, Ver. 20.2, Neurobehavioral Systems, USA) running on a Windows-based PC (Windows 7 Professional OS). The visual stimuli were projected via a LCD projector (EH-TW6600, Epson, Japan: 60-Hz refresh rate) onto a white screen hanging from the ceiling of the booth such that the display was 2.4 m in front of the participant. The video image was 66 × 44 cm (720 × 480 pixel) in size with a visual angle of 15.7 × 10.5 degrees. Auditory stimuli were presented via headphones (SHE9720, Philips, Netherlands) and the individual participants adjusted the sound intensity so that they could hear the music comfortably.

#### Materials

**Fig 1:**
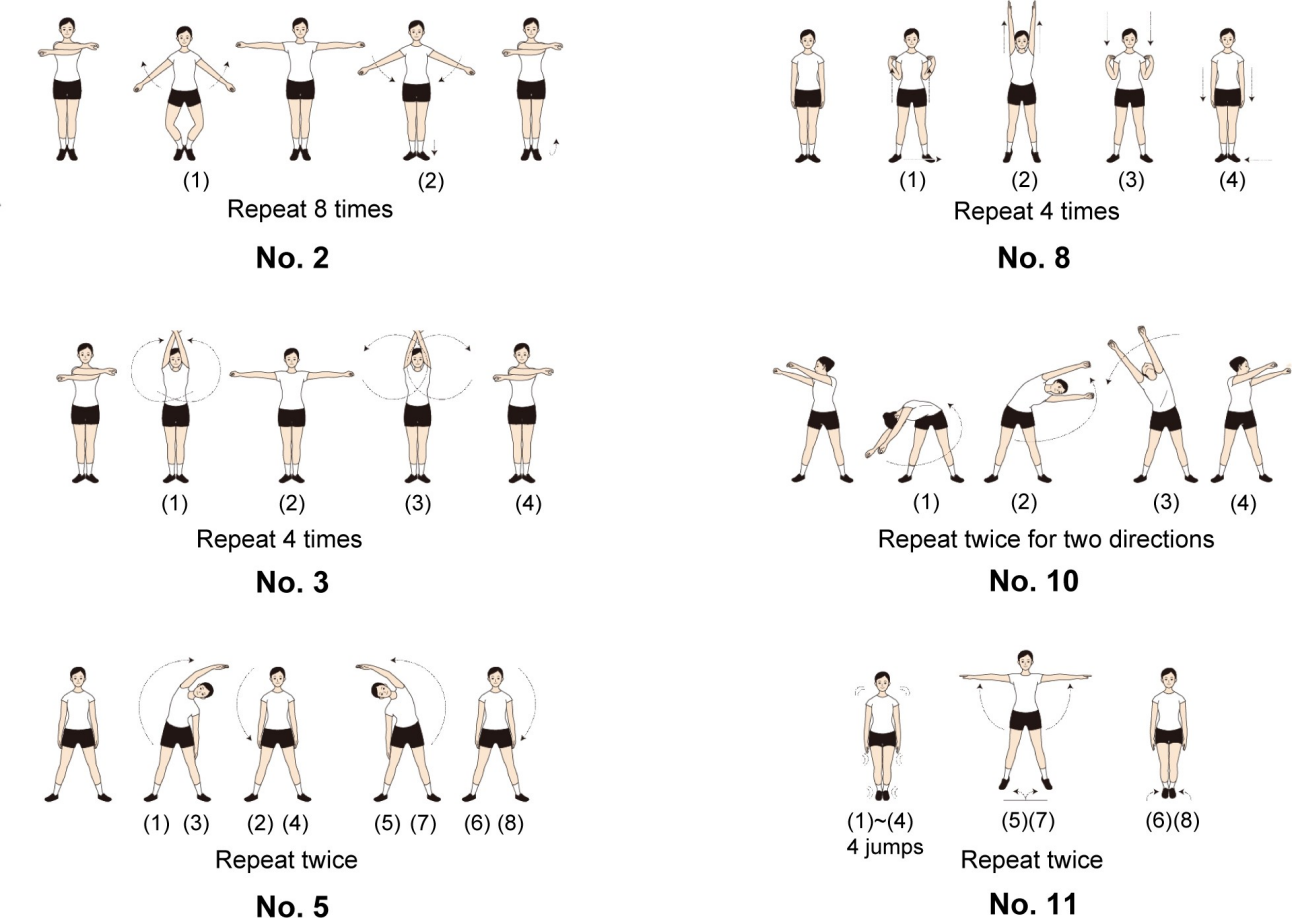
Exercises used in the experiments. This figure illustrates the six exercises from Radio Calisthenics routine No. 1 used in the present experiments. Numbers in parentheses indicate the approximate correspondence to the music beats. These illustrations are adapted from [67] with permission.

We generated video clips of an individual performing Japanese Radio Calisthenics routine No. 1 for the present study. We used a digital video camera (HC-V720M, Panasonic, Japan; 1920 × 1080 pixel resolution; 30 fps) to record an official Calisthenics instructor performing that routine in time to music generated by audio play software (Windows Media Player). We edited the obtained video clips with video-editing software (Vegas Pro13, Magix, Japan). We chose six exercises (#2, #3, #5, #8, #10, and #11) with 16 beats each (four bars with four beats in each) that consisted of two, four, or eight repetitions of the same action (note that the beginning and ending were modulated due to the *liaison* with the adjacent exercises). For diagrams of the exercises see Fig 1. In Exercise #2, “arm swing with knee bending”, a performer swings their two arms in outward and inward directions in front of their trunk while bending/stretching their knees. This action is repeated 8 times. Exercise #3, “arm rotation”, is similar to #2, but the performer fully rotates their arms without bending their knees. In Exercise #5, “lateral bending”, a performer raises their right arm above their head and bends their trunk and arm to the left at the 1^st^ and 3^rd^ beats, and raises their left arm above their head and bends to the right at the 5^th^ and 7^th^ beats. This set is then repeated. In Exercise #8, “arms extending in the vertical direction”, a performer first touches their shoulders with their hands (1^st^ beat), and then extends their arms upwards (2^nd^ beat). Then, they touch their shoulders again (3^rd^ beat) and extend their hands downwards (4^th^ beat). These actions are repeated four times. In Exercise #10, “upper body rotation”, a performer rotates their trunk, head and arms together in the left direction and then again in the right direction, and repeats these actions once. In Exercise #11, a performer jumps in place 16 times while extending his/her legs and arms outwards at the 5^th^, 7^th^, 13^th^, and 15^th^ beats. Sample video footage can be found on the home page of Japan Post Insurance Co. Ltd. [66]

The durations (including fade-in) of the video clips were 7 s (= 210 frames) for Exercises #2, #3, #5, #8, and #10 and 5 s (= 150 frames) for Exercise #11. The clips were cut from the full exercise routine, and included only 6 music beats. Note that, unlike previous studies that used regular beats such as a metronome sound, the beat intervals in the present study were not constant, but were altered according to the characteristics of the actions in each exercise. Specifically, the music tempo was faster for Exercise #11 (jumping) compared with the other exercises. Moreover, beat intervals altered even within a single exercise. Likewise, the visual stimuli contained some spatiotemporal fluctuations among different beats because we used a natural performance as the experimental stimuli.

We introduced audiovisual asynchrony (temporal shift) by changing the onsets of the sound and video streams using Presentation software [7]. To this end, we stored the sound and video signals separately in WAV and AVI file formats (the video encoder was DV). We applied a fade-in/out (2 s) at the beginning and end of the sound signal to prevent an abrupt onset from giving any unexpected clues regarding synchrony judgment. A fade-in (2 s) was also applied to the video signal.

We determined the range of the temporal shifts based on prior research [7,8,19] and then adjusted these values according to the results of a pilot experiment. Resultantly, we adopted −400, −300, −200, −100, 0, 100, 200, 300, 400, 500, and 600 ms as the temporal offsets (i.e., 11 conditions), where a zero value corresponded to the original video timing, and negative and positive values corresponded to audio-lead and audio-lag conditions, respectively. We expected that the asymmetry in the shift conditions was due to asymmetry in sensitivity to audiovisual asynchrony, as has been reported previously [8].

#### Procedure

Participants were seated on a chair in front of the screen while wearing headphones. Each trial started with a starting message, and a stimulus was presented about one second afterwards. The participants were asked to judge whether the performer’s body motion was synchronous or asynchronous with respect to the music, and to respond by pressing corresponding keys on a PC keyboard. Participants were expected to simply judge the synchrony between the music and body motion, instead of the simultaneity between the music beats and specific postures or actions (note that this is different from the procedure in [19]). The task was not timed, and if participants did not respond by 2 seconds after the end of video clip, the same stimulus was presented again (up to three times total). If the participant made a response during the second or third presentation, the stimulus was shut off immediately after the response.

Before starting the formal session, participants performed 12 familiarization trials where they were able to adjust the sound level so that they could hear the music clearly and comfortably. In the formal session, participants performed 110 trials (11 temporal shifts × 10 times) for each exercise, and the order of temporal shift conditions was pseudo-randomized. We divided the 110 trials into two blocks of 55 trials, separated by a short break. We ran the experiment for Exercises #2, #5, and #11 (total 330 trials = 55 trials × 2 blocks × 3 exercises) (set A) and that for Exercises #3, #8, and #10 (set B) separately. Ten participants took part in each experiment set such that 5 out of the 15 total participants took part in both sets. An experimental session took about one hour.

#### Analysis

We obtained a psychometric function by fitting the ratio of synchronous responses in the 11 shift conditions with a Gaussian function (*p*(*τ*) = α exp(−(*τ*−*τ*_0_)^2^/2*σ*^2^)), where *τ*_0_ is the mean, *σ* is the standard deviation, and α(≤1) is the amplitude. The point of subjective simultaneity (PSS) was defined by *τ*_0_ and the temporal integration window (TIW) was defined by the full width at half maximum (FWHF), which was related to *σ* by $FWHF=2\sigma \sqrt{2\log2}\approx 2.35\sigma$.

To conduct signal processing for the music element of the stimulus, we manually extracted the temporal beat position based on the energy distribution in the sound spectrogram. Beat timing could be determined without ambiguity because the piano sound had sharp energy onsets. We identified the video frames corresponding to the music beats using the video editing software.

Statistical tests were performed with a generalized linear model in R software [68].

### Results and discussions

**Fig 2:**
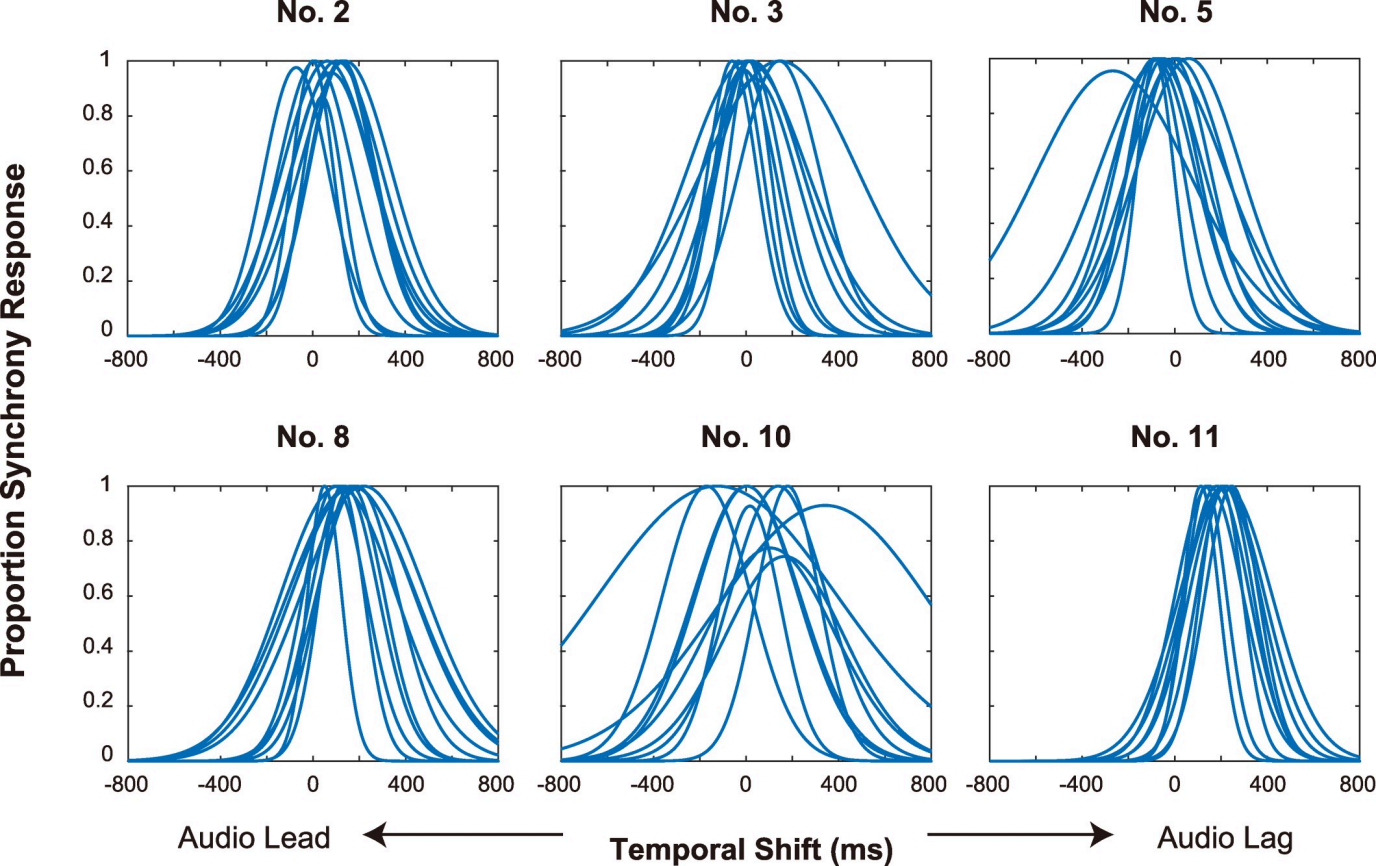
Perceived synchrony distributions in individual participants. Each curve indicates the estimated psychometric function for synchrony perception in an individual participant. These curves are located close to each other, indicating that different participants had similar synchrony perception during observation of the Radio Calisthenics routine.

**Fig 3:**
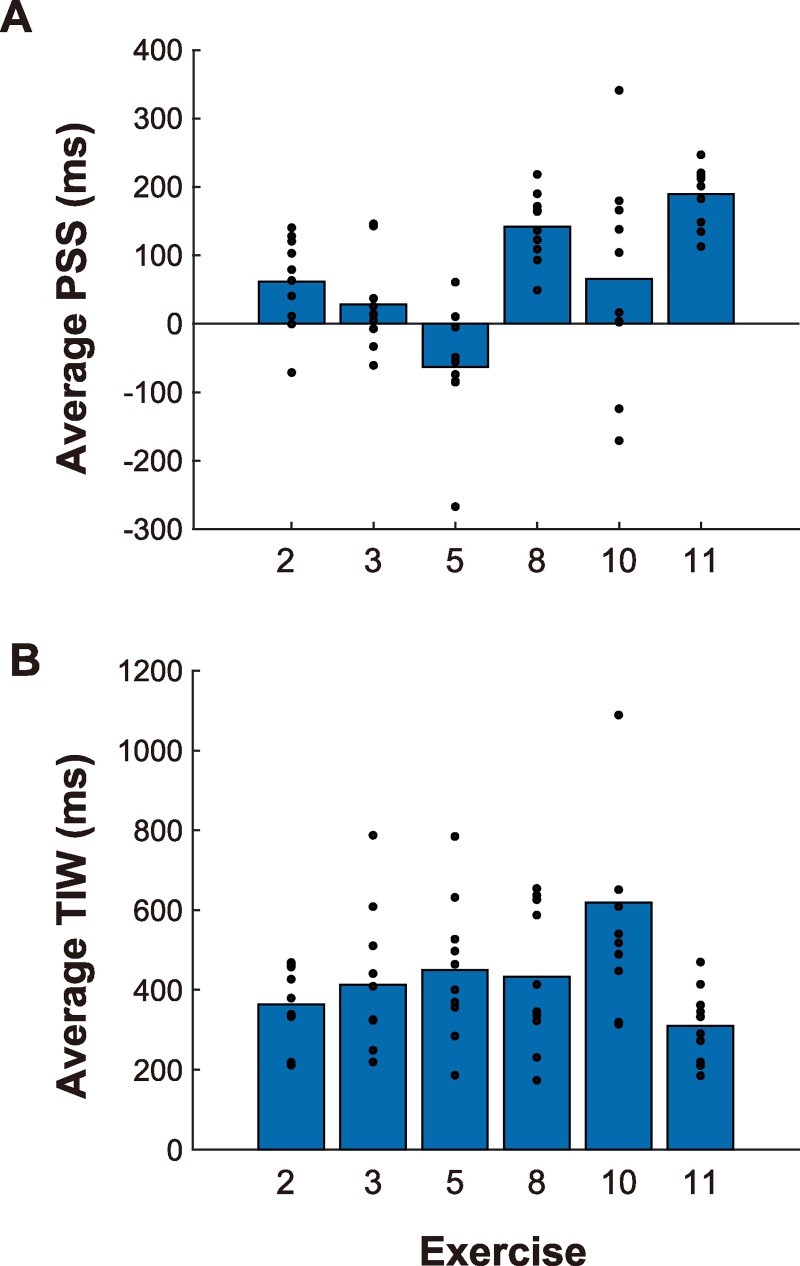
Point of subjective simultaneity (PSS) and temporal integration window (TIW). Panels A and B show the average PSS and TIW values, respectively. Small filled circles indicate the data from individual participants.

All participants completed the task and all data were usable. Fig 2 shows the individual psychometric functions, and Fig 3 summarizes the average PSS and TIW for each exercise, where small filled circles show the individual data. We briefly discuss the PSS and TIW before examining the movement feature points that coincided with music beats.

First, we found consistent PSS values among different participants. The range of PSS values in different participants was about 200 ms (except for in Exercises #5 and #10). This was smaller than TIW, indicating that all participants perceived synchrony at similar temporal-shift conditions. This result is important because there is no objective point of synchrony defined between the music and body motion of the performer. Thus, there appear to exist certain clues for judging synchrony that are common to all participants. Second, the average TIW was 300–450 ms (except for in Exercise #10), which is close to the values observed in previous studies (350–400 ms [8] and 330 ms (converted from *σ* to FWHF) [19]). This shows that the threshold for detecting audiovisual asynchrony is similar irrespective of whether the synchrony can be defined objectively or only subjectively, whether the visual stimulus is natural or a point-light figure, and whether the beat interval is regular or fluctuated. One exceptional case was Exercise #10, in which inter-participant variability in PSS and the average TIW values was larger (that is, the synchrony perception was more ambiguous). A statistical analysis using a generalized linear model ((TIW) ~ (exercise)) showed that the effect of Exercise #10 was significant (*p*<0.01). Presumably, this variability was due to the characteristics of this exercise: The performer rotated their trunk slowly using large movements to beat intervals that varied largely compared with the other exercises. These differences with respect to the other exercises might have made it difficult to judge synchrony.

Absolute PSS values seemed to vary somewhat among the exercises: Generally, the PSS was larger (i.e., audio-lag) for Exercises #8 and #11, but smaller (i.e., audio-lead) for Exercise #5 (lateral bending). Statistical analysis using a generalized linear model ((PSS) ~ (exercise)) showed significant effects of these exercises (*ps* < 0.01, 0.05, and 0.01 for Exercises 5, 8, and 11, respectively). At present, we have no clear explanation for these differences. In addition, PSS values were significantly larger than zero in Exercises 2, 5, 8, and 11 (*ps* < 0.05, 0.05, 0.001, and 0.001, respectively). The PSS has been found to be biased in the positive (i.e., audio-lag) direction, presumably due to asymmetry in sensitivity to audiovisual asynchrony [8]. However, we should note that absolute PSS depends on the temporal relationship between a performer’s body motion and music: If the performer’s motion incidentally lagged behind (or led) with respect to the music, this would modulate the absolute PSS. Further studies where PSS is estimated for multiple performances are needed to more comprehensively characterize this phenomenon.

**Fig 4:**
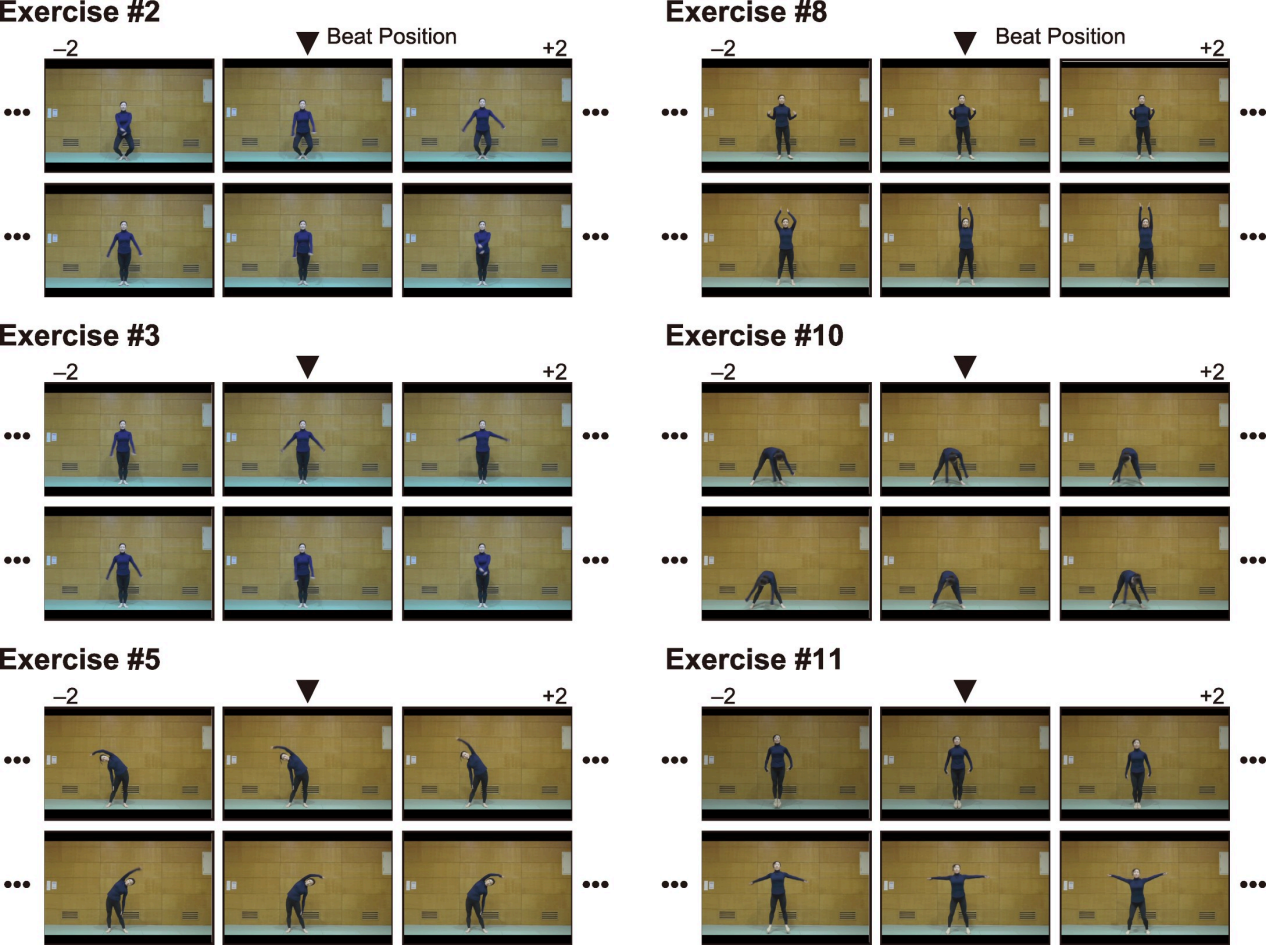
Movement features synchronized with music beats. This figure shows the video frames at the music beats when the audiovisual temporal shift was set to the average PSS value. In every panel, the central snapshot represents the frame at the music beat, and the intervals between adjacent snapshots are 2 frames (i.e., 66 ms).

Next, we describe the video frames that coincided with music beats when the temporal shift was adjusted according to the PSS. Fig 4 shows the video frames at the music beats for every exercise when the temporal shift was set to the average PSS value. The temporal interval between adjacent snapshots in the figure was 66 ms (= 2 frames). The detailed findings for each exercise are described in the following.

Exercise #2: The hands moved to the lowest (or bottom) position at the music beat. In the upper row, knees were bent almost maximally such that the upper body reached the bottom position.

Exercise #3: Similar to Exercise #2, the hands moved to the lowest position at the music beat, though the lowest position in the upper row was precisely two frames before the beat. Note that unlike in Exercise #2, the trunk position was maintained in this exercise.

Exercise #5: Lateral bending reached the endpoint at the music beat, though a slight temporal gap was observed (the endpoint was reached two frames before the beat) in the upper row.

Exercise #8: The hands reached the shoulders at the music beat in the upper row, and the arms were mostly stretched out at the music beat in the lower row.

Exercise #10: The arms moved through the bottom position at the music beat, as a component of a whole body rotation. A slight temporal gap was observed (the lowest position occurred two frames after the beat) in the upper row.

Exercise #11: The feet landed at the music beats.

In summary, two movement-feature points agreed with music beats: One was the lowest point of hand movement (Exercises #2, #3, and #10) and the other was the endpoint of the action (i.e., reaching and landing; Exercises #5, #8, and #11). Interestingly, movement initiation never coincided with the music beats.

The result that the lowest point agreed with the music beat seems to be consistent with previous findings. Su [19] argued that the bottom position of a bouncing movement was the visual reference point for a synchrony judgment because she had asked participants to judge the simultaneity of the lowest point and corresponding sound though her main argument was the significance of the peak velocity point. Similarly, the lowest point of a finger or baton movement appears to plays the same role in musical conducting [22]. Moreover, it also seems consistent with the finding that the visual information around turning-points of cyclic motion played a significant role in visuomotor coordination tasks [27–30]. One important difference between our findings and those of previous studies is the shape of the hand trajectory. In bouncing and conducting, the movement direction is reversed at the bottom position of movement. The bottom position is the turning-point of a back-and-forth motion (note that Luck [22] examined the effect of the curvature of the trajectory around the bottom position), and this salient feature plausibly serves as a visual reference point for synchrony. In contrast, the hand movements in Exercises #2, #3, and #10 are components of swing or rotation movements, and movement direction does not change at the lowest position; the hands just move “through” the lowest position. Thus, the way in which the lowest point could serve as a visual reference in these exercises is unclear. This question is discussed further below.

The movement endpoints were another feature point that was matched to the sound beat. The role of an endpoint is presumably similar to that in actions that produce a sound (e.g., hammering): The moment that the hand reaches the target position (e.g., hands touching the shoulders in Exercise #8 and feet landing in Exercise #11) serves as the reference point (like the point at which the hammer reaches the target).

In summary, the present result indicates that the participants perceived synchrony between the music and body motion when the lowest positions or endpoints of the movement of extremities (i.e., hand/foot) agreed with the music beats. It is important to consider that the body movements were continuous and (at least partly) predictable from the past trajectory, especially when the observer was familiar with the movement. Thus, an observer may rely on predictions to judge the synchrony between music and body motion. This would enable them to make successful judgments even when the reference points are invisible. Specifically, the brain may predict the timing of reference points based on preceding visual information and utilize it for synchrony judgment. The role of such predictions has already been discussed with respect to timing perception of auditory signals [19], but this was only for regular beats (like those produced by a metronome), and did not consider complex body movements.

To determine whether visual information about the lowest position and endpoint were indispensable for audiovisual synchrony perception, we conducted another experiment.

## Experiment 2

### Methods

#### Participants

Ten undergraduate and graduate students (8 men and 2 women) took part in this experiment. The other conditions were the same as in Experiment 1.

#### Apparatus

The experimental settings were the same as in Experiment 1.

#### Materials

**Fig 5:**
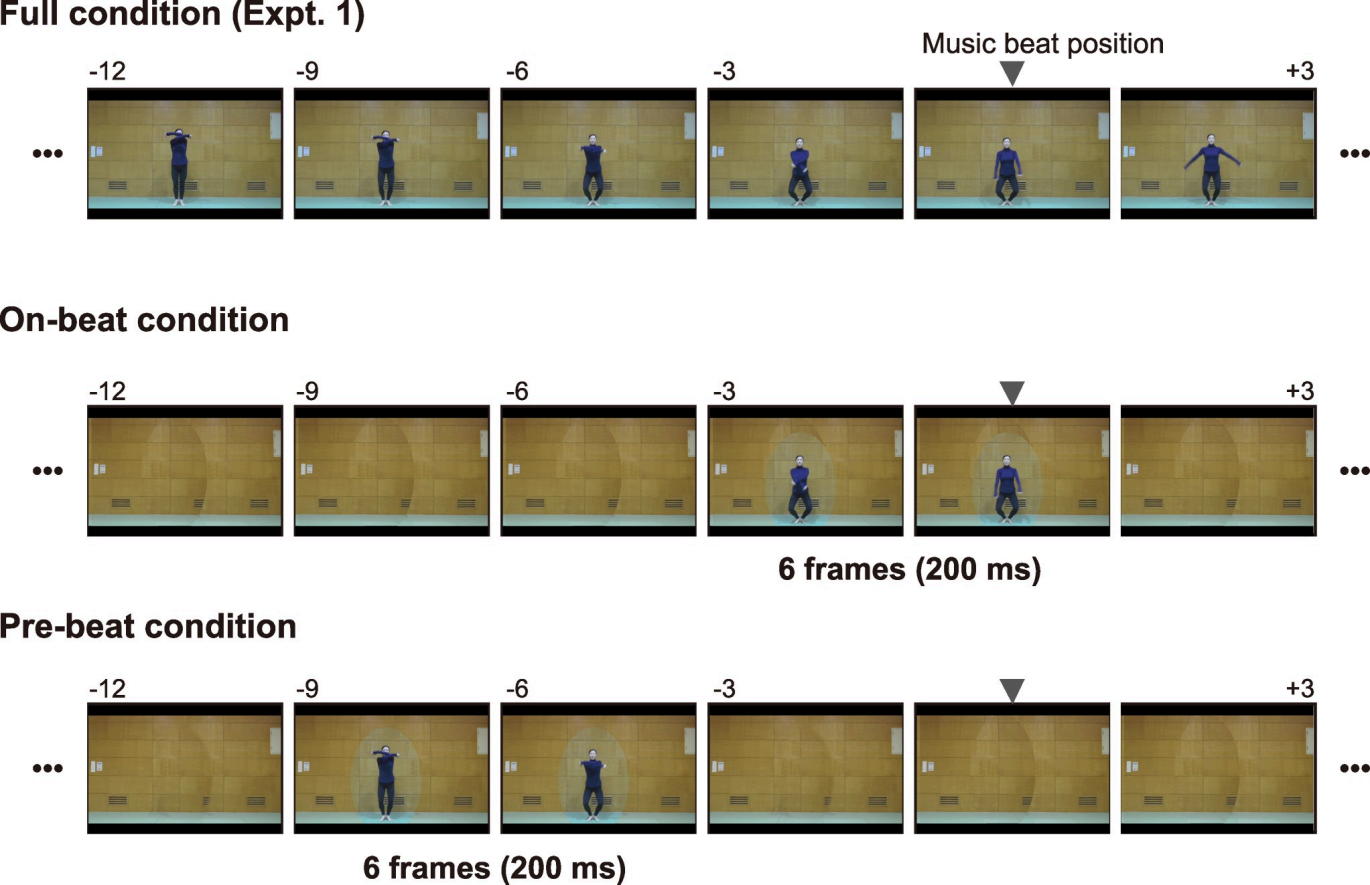
Visual stimuli used in Experiment 2. This figure shows the configuration of the visual stimuli used for Exercise #2 in Experiment 2. In the on-beat condition, visual information about the performer’s motion was provided only in six frames (= 200 ms) around the music beats. In the other frames, the region containing the performer was filled with a static background image. In the pre-beat condition, the performer’s motion was presented in the frames prior to the music beats. The intervals between the adjacent snapshots contain 3 frames (i.e., 100 ms). The visual stimuli for Exercise #8 were presented in the same manner.

In this experiment, we used two exercises (#2 and #8) containing different types of critical feature points (i.e., the lowest position and endpoints). The duration of the video clips was 7 s (= 210 frames) as in Experiment 1. To present only the movement of the performer that took place around the critical feature points, we extracted 6 frames (i.e., 200 ms) around the music beats. In the other frames, an elliptic region covering the performer was filled by a static image of the background of the scene so that the observers could not see the performer’s motion (Fig 5).

We prepared two conditions for the extracted temporal region. In one condition (“on-beat”), the visual information about the performer’s motion was presented in the frames that included the music beats when the temporal shift was set to the average PSS obtained in Experiment 1. We chose these frames so that the music beat would be located around the 3^rd^ and 4^th^ frames in the six-frame region of the average PSS data. In the other condition (“pre-beat”), the performer’s motion was presented in the frames prior to the music beats. The selected frames in the “pre-beat” condition were the 7 frames (i.e., 233 ms) prior to those in the “on-beat” condition (Fig 5).

#### Procedure

The procedure was similar to that of Experiment 1. The total number of trials was 440 (= 110 trials × 2 conditions × 2 exercises) divided into 8 blocks of 55 trials. The experiments for Exercise #2 and for Exercise #8 were run in separate sessions.

### Results and discussions

**Fig 6:**
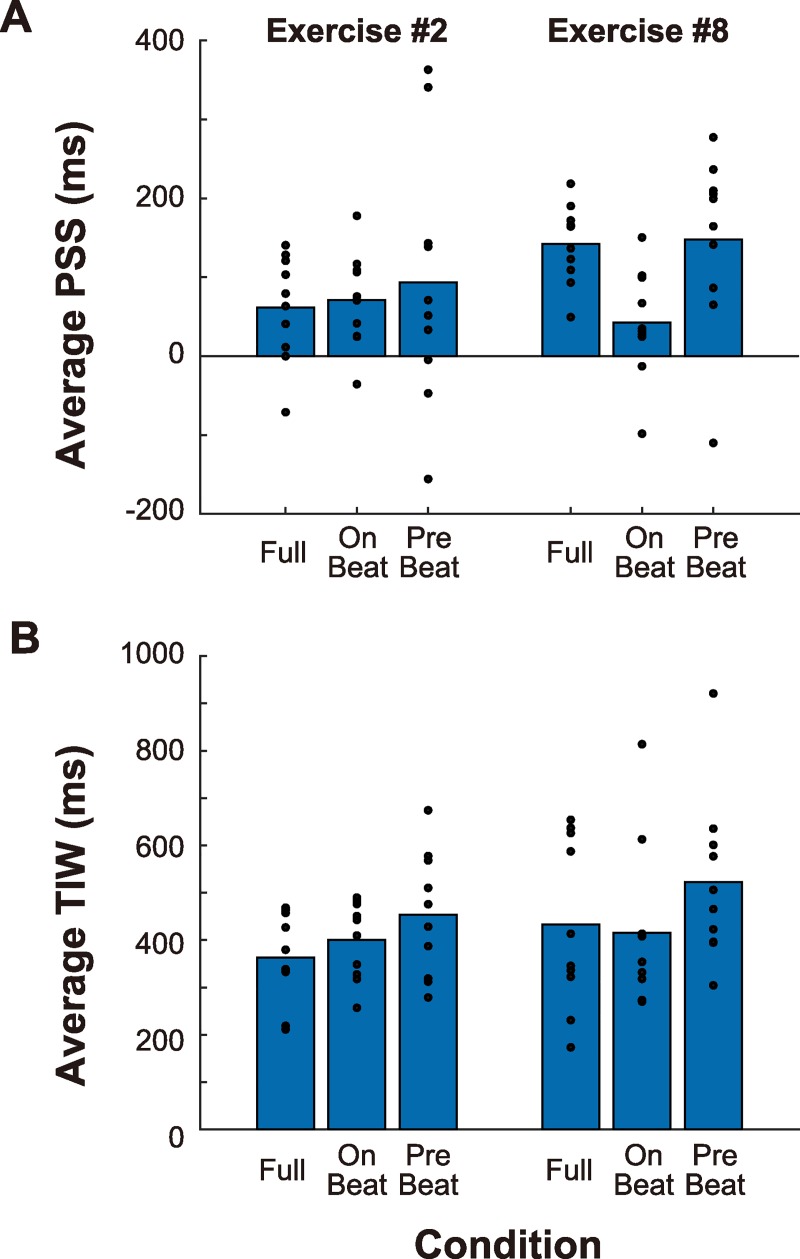
Point of subjective simultaneity (PSS) and temporal integration window (TIW) values in Experiment 2. Panels A and B summarize the average PSS and TIW values, respectively, estimated from the psychometric functions in Experiment 2. Small filled circles indicate data from individual participants.

All participants completed the task, and all data were useable. Fig 6 summarizes the average PSS and TIW values in the two conditions, together with those obtained in Experiment 1 (i.e., full condition). Small solid circles show the data from individual participants. First, the PSS values for Exercise #2 were nearly the same in all trials, irrespective of condition, but the PSS values for Exercise #8 were much smaller in the on-beat condition, compared with that in the full and pre-beat conditions. A generalized linear model ((PSS)~(exercise)*(condition)) showed quasi-significant coefficients of the effect of exercise (*p* = 0.0628) and that of the interaction between Exercise #8 and the on-beat condition (*p* = 0.0745). However, the TIW in the pre-beat condition tended to be longer than that in the on-beat condition. A generalized model ((TIW)~(exercise)+(condition)) showed a quasi-significant effect of the video condition (*p* = 0.0502).

Our data indicate that the participants were able to consistently perceive synchrony in music and body motion even when the movement features at the music beats were invisible, suggesting that some prediction mechanism is involved in synchrony perception. However, the average TIW (and PSS variance) was larger in the pre-beat condition. This is presumably because the internal prediction was not perfect, leading to ambiguity when visual information about the critical feature point was lost. Interestingly, in Exercise #8 we found that the PSS in the full-condition (i.e., Experiment 1) was closer to that in the pre-beat condition rather than that in the on-beat condition. That is, the judgment in the case where all of the video frames were visible differed from that in the case where only feature points on the beats were visible, such that it was similar to that in the case where feature points were invisible. This suggests that our brain makes use of internal prediction preferentially, even when visual information is fully available.

In summary, the results of Experiment 2 indicate that the synchrony between music and human motion was steadily perceived when the participants could not see the visual reference points matched to the sound beats. This demonstrates that direct comparison between visual and audio reference points is not required for audiovisual synchrony perception. Thus, our perceptual system may make use of preceding sensory information to anticipate the timing of reference points.

The results of Experiment 2 suggested that internal predictions are engaged in synchrony perception. We examined this finding more comprehensively in the next experiment.

## Experiment 3

### Purpose and rationale

The results of Experiment 1 led us to ask why the lowest positions of hand movement in Exercises #2, #3, and #10 served as visual feature points that were matched to the sound beat. As mentioned earlier, these exercises contain no rhythmic vertical motion such as that observed in bouncing and conducting movements. In addition, unlike the turning point in bouncing and conducting movements, the hand (arm) movement around the lowest position is generally a component of continuous rotation, and hardly gives an impression of a “visual beat”. Thus, the way in which the lowest position serves as the reference point is unclear. To address this issue, we considered two viewpoints.

The first perspective concerns the coordination (or synergy) of human body motion. It is broadly accepted that body parts move in a coordinated manner to accomplish a given motor task[57,58], and naturally, this is true for physical exercises. So far, we have considered only the movement features of local body parts (such as the lowest position of the hand during movement) as feature points. With respect to whole body coordination, however, we should also consider the spatiotemporal characteristics of global variables that reflect body movement as a whole.

The second perspective concerns the “common coding of action and perception” [32–34] and “mirror system” [35–39] theories, which suggest that there is a close relationship between perceptual and motor representations in the brain. According to these theories, seeing an action activates the motor representation associated with that action, and performing an action activates the perceptual representation associated with that action. If we accept this view, then it follows that the neural representation for a motor action (and/or perception during its execution) is activated in an observers’ perceptual system while viewing a motor performance.

Generally, it seems reasonable to assume that a person engaged in an exercise (performer) intends to synchronize specific actions to the beat of the music. In Exercise #8, for example, the performer intends to touch their shoulders with their hands and to stretch out their arms in accordance with the beat timing. In Exercises #2 and #3, however, it is unlikely that performers consciously intended to synchronize the lowest positions of hand motion with the beat (no participants mentioned in the post-experiment informal discussion that they noticed it). Therefore, the feature points matched to the music beats are not necessarily considered “reference points” in a conscious manner.

Then, what is the meaning of the lowest hand position for the performer? Considering the first viewpoint, it may be helpful to examine global variables representing body motion as a whole, in addition to the motion of individual body parts. A single global variable may have feature points that are related to those of local body parts.

To test this idea, we focused on the total external force acting on the body as a global variable that can be monitored via the ground reaction force (GRF). If we regard the whole body as a point mass, the external forces acting on the body are gravity force and GRF. Because gravity is constant, only GRF can vary temporally. Thus, GRF can be used to represent whole body dynamics. Moreover, individuals are able to sense GRF as a tactile stimulus on the sole of the foot. When dancers (especially street dancers) move their trunk in a vertical direction to rhythmic music by bending/stretching knee joints [49–51], vertical GRF (vGRF) (and foot sole sensation) varies rhythmically given that it is synchronized with the beat of the music. However, we do not know whether the vGRF behaves similarly during physical exercise to music. Thus, in Experiment 3, we first examined the temporal behavior of vGRF during Radio Calisthenics. Second, we measured the temporal correlation between motion in the extremities (hand/foot) and changes in vGRF. If these are tightly coupled, then we can think that vGRF (or foot sole sensation) may be an essential variable in timing perception for physical exercise. Furthermore, according to common coding theory, observers might anticipate the sensations that accompany the actions of the performer in the video image and perceive synchrony based on the temporal relationship between the music beats and the anticipated sensation.

We conducted Experiment 3 to examine the validity of this hypothesis. We measured body motion and GRF as individuals performed Radio Calisthenics, and analyzed the temporal relationships between body motion, vGRF, and musical rhythm.

### Methods

#### Participants

Three graduate students (3 men) took part in the experiment. They had all participated in Experiments 1 and 2.

#### Apparatus

We used an optical motion capture system (Optitrack Prime13, Natural Sense, USA; 6 cameras, 240 fps) to record the 3D trajectory of the body movement of the participants. Twenty-eight reflexive markers were attached to the head, neck (cervical spine C7), waist (L5), shoulders (acromion), elbows (lateral epicondyle), wrists (ulnar/radial styloid process), fingertips (middle finger), great trochanter, knee joints and foot joints (lateral malleolus), and heels of the participants.

Ground reaction force (GRF) was measured via two force plates (MF-4060, Tech Gihan, Kyoto, Japan) with a sampling rate of 1000 Hz. The data acquisition was synchronized with the motion capture system using an external trigger signal. The music stimuli were played on a PC (Windows 7 Professional OS) with audio software (Windows Media Player) and presented via a single-channel loud speaker (Yamaha, MSP-3, Hamamatsu, Japan). The sound signal was recorded only in the left channel, and a timing signal (pulses with 10-s intervals and 10-ms width) was recorded in the right channel of a WAV file. In the experimental trials, we played the music from the left-channel signal and turned on/off an infra-red LED from the right-channel signal so that the timing signal was captured by the motion capture camera. Afterwards, we corresponded the motion data to the music using these timing markers.

#### Procedure

Before starting the experiments, an experimenter attached the reflexive markers to the body of each participant using adhesive tape. Then, the participants performed Radio Calisthenics routine No. 1 on two force plates (mounted adjacently). They repeated the performance five times, with short breaks between the trials. The session took about 40 minutes, including preparation.

#### Analysis

We used Exercises #2, #3, #5, #8, and #11 for this analysis. The lowest position coincided with music beats in Exercises #2 and #3 and the endpoints coincided with music beats in Exercises #5, #8, and #11. Although we obtained a variety of data from the motion capture system and force plates, we focused our analysis on the vGRF, left wrist, left foot-joint, and neck (spine C7). We applied a low-pass filter (4^th^-order Butterworth filter) to the motion capture data (cut-off frequency: 10 Hz) and GRF data (cut-off frequency: 50 Hz). Then, we extracted specific movement feature points (i.e., the lowest position of wrist motion (Exercises #2 and #3), the endpoint of wrist motion (Exercises #5 and #8) and the endpoint of foot-joint motion (Exercise #11)) and the local peaks of vGRF using Matlab software (Mathworks, USA). Specifically, the lowest points were automatically extracted using the “findpeaks” function in Matlab and the endpoint was determined based on the point with the lowest velocity in the wrist and foot-joint movement data. We examined the extracted peak positions and applied corrections manually when the wrong peaks were detected. We excluded data at the 1^st^ and 16^th^ beats from the statistical analysis because the body motion around these beats was distorted due to the *liaison* with the adjacent exercises. We also excluded data at the 4^th^, 8^th^, and 12^th^ beats for Exercise #5 because we could not find clear vGRF peaks around these beats.

### Results and discussions

We obtained 15 datasets (3 participants × 5 trials), although the motion capture data from one trial was collapsed. Resultantly, we analyzed data from 14 trials.

**Fig 7:**
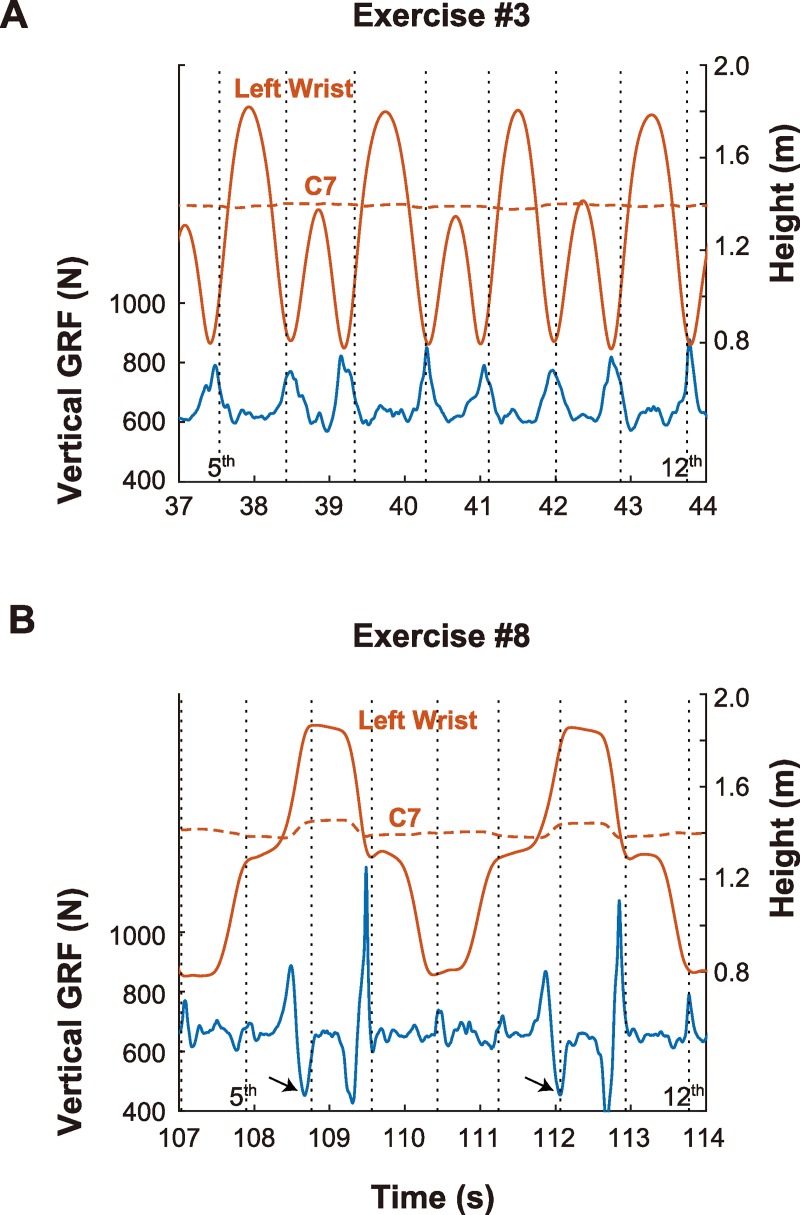
Temporal relationship between vGRF and body motion. This figure shows two examples of temporal changes in the vertical ground reaction force (vGRF) and in the heights of the left wrist (solid line) and spine at C7 (broken line). Panels A and B shows the data from Exercise #3 and #8, respectively. Vertical broken lines indicate the timing of music beats (from 5^th^ to 12^th^). Generally, temporal locations of the vGRF peaks coincided with the music beats. Small arrows indicate the cases in which the negative vGRF peaks coincided with beats. These vGRF peaks has good agreement with the timings of the lowest positions (Exercise #3) and endpoints (Exercise #8) of wrist movement.

First, we examined the temporal profiles of the vGRF data and the position of the left wrist and neck. Fig 7 shows two representative datasets obtained from a single trial with one participant. The upper and lower panels show the results for Exercises #3 and #8. In each panel, the blue solid curve shows the vGRF, and the solid and broken orange curves indicate the heights of the left wrist and neck, respectively. Vertical broken lines indicate the timing of the music beats.

First, we examined the data from Exercise #3 (Panel A). As we found in Experiment 1, the temporal location of the lowest points of the wrist coincided with those of the music beats, although we observed some fluctuations. In addition, we observed periodic sharp peaks in the vGRF and the timings of these peaks agreed with the music beats. The behavior of the vGRF was unexpected because this exercise was not accompanied by overt vertical movement, as can be confirmed by the fact that the neck height was maintained throughout the exercise. We suppose that this result was due to the reaction in the torso to the centripetal force generated by arm rotation. Specifically, the vGRF increased as the hands descended; this was likely due to the increase in the angular velocity of arm rotation and the directional change in the centripetal force (which became vertical when the hands reached the bottom position). Further examination indicated that the timings of both the vGRF peaks and the lowest positions fluctuated around the music beats, and that their distance from the music beats was well correlated, indicating tight coupling between vGRF and wrist behavior.

Next, we examined the data from Exercise #8. The hand movement in this exercise consisted of four segments: 1) the hand moves up to the shoulder; 2) is raised in an arm stretch; 3) moves back to the shoulder, and 4) returns to the original lower position. The timing of the end of each segment was in approximate agreement with the music beats. Although the temporal behavior of the vGRF was rather complicated compared with that in Exercise #3, the timing of the local peaks coincided well with the music beats. Interestingly, not only the positive peaks but also the negative peaks coincided with the music beats. The vGRF had negative peaks at the 2^nd^, 6^th^, 10^th^, and 14^th^ beats (as indicated by small arrows in the figure) and positive peaks at the other beats. Presumably, these negative peaks were caused by rapid deceleration of the arm stretch movement. Actually, the temporal positions of the negative vGRF peaks were close to the maximum deceleration points of the wrist movement (not shown in the figure). Similarly, the positive sharp peaks at the 3^rd^, 7^th^, 11^th^, and 15^th^ music beats were due to the deceleration of the arm drop movement. Therefore, these vGRF peaks were not linked to the movement endpoint but to the peak deceleration, according to the principles of Newton mechanics. Thus, in terms of the potential role of a global variable represented by vGRF, the maximal deceleration point (instead of the movement endpoint) may be appropriate as the reference point. We discuss this point further below.

As pointed out in the Introduction section, previous studies have shown significant contribution of the vestibular system to rhythm perception [40,41,59–64]; it is plausible that the vestibular signal may represent the timing of whole body motion because most dance movements are accompanied by vertical trunk movements in synchronization with music beats [49–51]. However, this does not seem true for the exercises examined in this experiment. As can be seen in Fig 7(A), the neck height was almost maintained throughout Exercise #3, suggesting that the vestibular system were hardly activated in this exercise. Additional analysis of the head motion (using the motion capture data of markers attached on the participants’ head) showed that head acceleration showed many local peaks asynchronously with music beats though some of the peaks coincided with the vGRF peaks. Thus, we conclude that vestibular input did not play a significant role in the present experiment although we cannot completely rule out the possibility that the vestibular system is involved in the synchrony perception between music and body motion.

**Fig 8:**
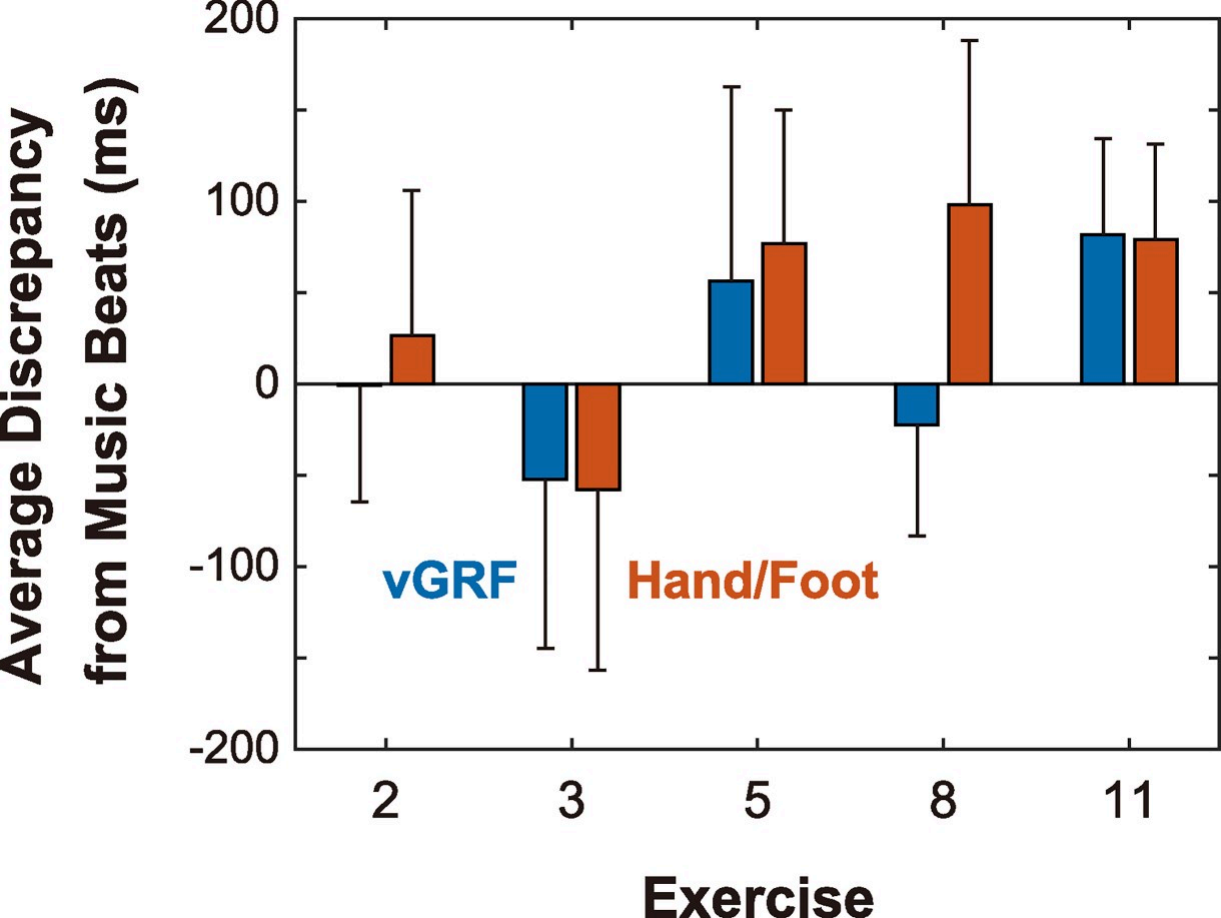
Temporal displacement between peak vGRF, movement feature points, and music beats. This figure shows the average temporal displacement between vGRF peaks and music beats and that between the feature points of hand/foot motion and music beats. Error bars represent the standard deviations.

We statistically tested the above observations. First, we estimated the relative distance from the music beats for the vGRF peaks and movement feature points. The results are summarized in Fig 8. Generally, the mean temporal discrepancy was less than 100 ms, meaning that the feature points almost agreed with the music beats, although statistical analysis showed that these differences were significantly larger than zero in most exercises (except for the difference between the GRF peaks and music beats in Exercise 2). Moreover, these two discrepancies were closely correlated. The correlation coefficients for exercise #2, #3, #5, #8, and #11 were 0.594, 0.948, 0.686, 0.441, and 0.811, respectively, and all were highly significant (*p*s < 0.001). This shows tight coupling between vGRF peaks and the feature points of motion of the extremities.

Two notable patterns are visible in these data. First, for Exercise #8, the mean discrepancy from the music beats was remarkably different (by 120 ms) between the vGRF data and the wrist motion, although it was almost the same for the other exercises. Statistical analysis using a generalized linear model ((Discrepancy) ~ (GRF or Body)) (performed separately for individual exercises) supported this view (*p* < 0.001), although significant effects were also detected for Exercises #2 and #5 (*p*s < 0.001 and 0.05). The large discrepancy found in the data from Exercise #8 appears to have been caused by the disagreement between the maximal deceleration point and the movement endpoint, as pointed out above. During the arm raise/drop movement, the maximal deceleration, which is coupled with vGRF peaks, inevitably precedes the movement endpoint, causing the discrepancy, as noted by Luck and Sloboda [22]. Actually, the temporal gap between the maximal deceleration point and the endpoint was about 100 ms, which is close to the above difference (120 ms). That the relative difference from the music beats was smaller for the vGRF suggests that the performer intended to synchronize the vGRF peaks to the music rather than to the movement endpoint. In the other exercises, the vGRF peaks and movement features are physically linked, and thus, their timings are in agreement.

Second, the relative differences from the music beats were remarkably larger than zero in Exercise #11. In examining the vGRF temporal pattern in detail, we found that the music beats were located between the vGRF onset (i.e., the moment at which the toes touched the ground) and the vGRF peak (i.e., the moment at which the whole body reached the lowest position). In other words, the performers touched the ground ahead of the beat, and reached the bottom position behind the beat. At present, we are unable to determine which point agreed with the music beats because of inter-participant difference. However, our data indicate that the characteristic points (i.e., rapid onset or maximal peak) of the vGRF were synchronized with the music beats.

Therefore, we found that the vGRF peaks consistently were synchronized with the music beats irrespective of the type of exercise. In contrast, the different features of motion of the extremities were synchronized with the music beat depending on the exercise type. These findings suggest that the total force acting on the whole body (or the sensation at the foot sole) is more tightly coupled with the music beat rather than the motion of individual body parts. The motion of local body parts produces a reaction force that acts on the body, and temporal changes in this force appear in the vGRF data. Considering that the vGRF can be sensed as a tactile stimulus at the foot sole, one tentative but interesting hypothesis is that synchrony perception during the observation of human motion to music arises from anticipated foot sole sensations given the visual information from the performer. Therefore, hypothetically, the reference point may indeed be not the lowest hand position itself but a feature point of a global variable co-occurring with the lowest hand position, for example, a reaction force acting on the body.

## Summary and general discussion

### Perception of synchrony between music and human motion

In the present study, we investigated the fundamental mechanism of audiovisual synchrony perception during observation of the human body in motion to music. We investigated movement features that were synchronized with music beats when participants perceived synchrony between music and body motion, with the Japanese Radio Calisthenics program as an example of motion accompanied by music. In the experiments, we asked participants to judge the synchrony between the music and body motion, and introduced a range of temporal shifts between the video and sound signals. We estimated the point of subjective synchrony (PSS) and determined the video frames that corresponded with the music beats for which the participants perceived synchrony.

An essential point of synchrony perception between body motion and music is that the synchrony cannot be defined objectively, but only determined subjectively. This was pointed out by Su [19], as mentioned in the Introduction section. However, our stimuli differed from her stimuli in several aspects, that is, we used natural video images while she used point-light figures, we used real piano music while she used controlled percussion sound, and we used complicated whole body movements while she used simple bouncing movement. In addition, the task direction was different: In Experiment 1, we asked participants to judge the synchrony between the music and body motion while she asked participants to judge the simultaneity between a percussion sound and the lowest position of a bouncing movement. Irrespective of these differences, the general results of the two studies were similar. Specifically, for Exercises #2, #3, and #10 used in the present experiment, the lowest positions of arm movement coincided with music beats. Although Su’s main claim [19] was the significance of the velocity peak, the lowest point and velocity peak point were close to each other and she originally proposed that the lowest point would correspond to the sound. Therefore, the present result appears to be consistent with her finding. As for the other exercises (Exercises #5, #8, and #10), the endpoints of the hand and foot movements coincided with music beats. Generally, the latter type of result was observed for goal-oriented actions (i.e., touching the shoulders, stretching out the arms, and landing from a jump) while the former type was observed when the movement was continuous (i.e., arm swing and rotation). Interestingly, the movement start point never coincided with the music beat. At present we are unable to explain this, although our data indicate that movement initiation is not important for synchrony perception during observation of human motion.

We estimated PSS and TIW values from the psychometric function of synchrony judgment. First, the mean TIW was about 300–440 ms, which nearly matched the values reported in previous studies (350–400 ms [8] and 330 ms [19]). Importantly, TIW did not vary according to whether the synchrony could be defined objectively (e.g., speech and drumming) or only subjectively (e.g., dancing). If we assume that synchrony perception is achieved by comparing visual and auditory reference points, this result implies that our brain might define some subjective “reference point” in the observed movement. This subjective reference point is clearly designated by Luck and Sloboda [22] as the “visual beat” in conducting movements. We will discuss this point below. Second, the PSS approximated zero for some exercises, but not for others. A non-zero PSS may reflect the existence of a discrepancy between the performer’s feeling of synchrony and the observers’ perception of synchrony. However, considering that the timing of the performer’s movement fluctuated trial by trial, the absolute value of PSS may change with other video data. At the present, we will not discuss this point further. To examine the dependency of PSS on video images, further experiments are necessary.

### Role of prediction in synchrony perception

As discussed in the previous section, the finding that specific movement feature points agreed with music beats during synchrony perception in Experiment 1 indicates that these feature points may serve as “visual references” to be compared with auditory reference points. However, considering that human body motion is continuous, it may be possible to anticipate the timing of visual references from stimuli encountered prior to the reference point, with the help of an internal forward model. Therefore, prediction may play some role in synchrony perception during observation of natural human movement.

We designed Experiment 2 to test this view. In this experiment, we restricted the visual image of the performer to limited temporal regions. In the on-beat condition, the performer’s motion could be seen only around the music beats, and in the pre-beat condition, the performer’s motion could be seen only prior to the beats (i.e., the reference points were invisible). We found that participants could judge the synchrony in both conditions, indicating that, consistent with our hypothesis, the visual image of the reference point is unnecessary for audiovisual synchrony perception. More precisely, the TIW was larger in the pre-beat condition, which implies that the internal prediction may not be perfect. This could lead to some ambiguity in synchrony judgments. The PSS for Exercise #2 was maintained under different conditions (including the full condition (i.e., Experiment 1)), indicating that the estimated timing of the reference point was unbiased. Intriguingly, the PSS for Exercise #8 was the same in the pre-beat and full conditions, but was smaller in the on-beat condition. In other words, synchrony perception in the natural viewing condition (i.e. full-condition: Experiment 1) was closer to that in the case in which the reference point was invisible (i.e., pre-beat condition) rather than the case in which only the reference point was visible (i.e., on-beat condition). This indicates that even in the natural viewing condition, the participants relied on internal prediction to judge synchrony between music and body motion.

In summary, the results of Experiment 2 suggest that internal prediction is involved in synchrony perception. This is a novel finding of the present study.

### Local isolated features vs. global coordinated features

Up until this point, we have assumed that the movement features of local body parts (such as hands) serve as visual references. Indeed, the motion of the hands (or arms) appears to be a very salient element of the exercise movement, and thus is likely to be a feature point that is important in synchrony perception. However, the lowest point in the rotation movement in Exercises #2, #3, and #10 to be minimally salient, and did not expect this position to give an impression of “visual beat.” An explanation for this may relate to the “coordination” or “synergy” view of human motor control [57,58]. That is, it may be necessary to consider the characteristics of body movement “as a whole” in addition to those of isolated local parts. Another viewpoint relates to the tight coupling between perceptual and motor representations, as proposed by the “common coding theory” [32–34] and “mirror system” [35–39] theory. According to these models, individuals anticipate motor intention (and/or sensation/perception during motor execution) when observing another person perform a body motion. Therefore, people may determine synchrony between body motion and music according to anticipated intention or sensation. This view seems attractive in that it offers a unified theory that explains synchrony perception during the observation of human actions. However, it does not explain why, in the present study, we found that the lowest position of arm rotation served as a reference point despite a lack of intention on the part of the performer to synchronize their movement in such a way.

Based on these considerations, we decided to examine the total force acting on the performer as a global variable reflecting the dynamics of whole body movement. Specifically, we analyzed the temporal behavior of the GRF. Because external forces acting on the body are gravity and GRF, and only GRF varies temporally, we used GRF to represent whole body dynamics. Moreover, GRF can be sensed as a tactile stimulus on the sole of the foot, and thus, our brain can use this information when monitoring the motion of our bodies. If we accept the view of common-coding theory, it is possible that the observers anticipated the sensation accompanied by the performers’ motor execution (i.e., vGRF) shown in the video and perceived synchrony based on the temporal relationship between the music and the anticipated sensation.

Although this general hypothesis cannot be tested by a single experiment, we conducted Experiment 3, in which we examined the temporal relationship between GRF, the feature points of local body parts, and music beats.

Resultantly, we found that in most exercises, vGRF periodically showed local peaks with temporal positions that coincided with music beats. This supports the possibility that vGRF peaks are potential reference points that are matched to sound beats. For the remaining exercises, we found that vGRF did not change periodically, but produced local peaks at the music beats. We also found that the temporal behavior of vGRF was significantly correlated with local body movements. As discussed in the section describing Experiment 3, this correlation can be explained in terms of Newtonian mechanics. Specifically, the local peaks of vGRF were caused by the centripetal force of rotation movement (Exercise #2, #3, and #10) or by the rapid deceleration of local body parts (Exercise #5, #8, and #11).

Therefore, our data indicate that the vGRF peaks were tightly coupled with the feature points of local body parts. Moreover, the vGRF peaks were consistently aligned with the music beats in different exercises while the local movement features that were matched to music beats depended on the exercise type. These findings indicate that “movement as a whole” is an essential factor in moving to music. Thus, an observer’s perceptual system may extract reference points from visual images of a performer and use these for synchrony judgment. In this sense, audiovisual synchrony perception during observation of human action might involve body sensations.

At present, this view remains hypothetical, and our present attempt represents the start of an investigation regarding this view. We plan to conduct further systematic experiments focusing on this issue. We may use point-light figures to examine the effect of the removal of specific point-lights on synchrony perception. This would enable us to directly test the significance of feature points for individual body parts. We proposed that our brain can anticipate the behavior of global variables from visual images. If this is the case, what visual information is utilized for anticipation? Are only local salient feature significant, or are more abstract global motion features essential? These questions could be tested by an experiment using point-light figures. Further, it would be interesting to examine the relationship between performers and observers. For instance, PSS and TIW values might differ in cases in which participants observe video clips of their own performance vs. that of others. If the mirror system is involved in synchrony perception, then observation of one’s own performance might decrease PSS closer to zero and decrease TIW overall. We believe that the two viewpoints (i.e., whole body motion and mirror system) discussed in the present article will provide new directions in the field of synchrony perception.

### Performing arts and synchrony perception

The ultimate aim of the present study was to understand the role of the temporal characteristics of body motion in dance performance. Because a dancers’ artistic message is conveyed by the motion of their body, the spatiotemporal characteristics of their body motion can serve as a medium for communication between the dancers and audience. Audiences expect performers to “dance to music”, that is, to synchronize their motion to the rhythm of the music. However, if dancers simply moved their body regularly to match music beats, their performance might appear dull. Presumably, dancers alter the timing of their actions to produce various impressions. Such alternations might serve to modulate the synchrony between the music and body motion. Specifically, dancers might adjust (even unconsciously) the discrepancy between the reference feature points and music beats. Another possibility is that dancers adjust the relative timings of motions of different body parts while maintaining a global motion that is synchronized with music beats. It is also possible to represent different levels of rhythm pattern by distinctively using multiple body parts. Actually, it was reported that different body parts were used to exhibit different levels of metrical hierarchy in some professional dancers [55,69]. The Radio Calisthenics program used in the present study is not a dance, and thus, we did not deal with aspects of artistic body expression. Future investigations might examine relationships between the spatiotemporal characteristics of body motion and artistic expression to assess the perceptual mechanisms involved in appreciating the performing arts.
